# Haematological manifestations of COVID‐19: From cytopenia to coagulopathy

**DOI:** 10.1111/ejh.13491

**Published:** 2020-08-31

**Authors:** Charles Agbuduwe, Supratik Basu

**Affiliations:** ^1^ University College London London UK; ^2^ University of Wolverhampton Wolverhampton UK; ^3^ New Cross Hospital Wolverhampton UK

**Keywords:** blood count, coagulopathy, COVID‐19, cytokine storm, D‐dimer, lymphopenia, SARS‐CoV‐2

## Abstract

Emerging data from the management of patients with coronavirus disease 2019 (COVID‐19) suggests multi‐systemic involvement, including the hemopoietic system. The haematological manifestations of COVID‐19 include blood count anomalies notably lymphopenia and neutrophilia which are of prognostic significance. Hyperferritinemia and elevated lactate dehydrogenase have also been associated with increased mortality. Furthermore, there is considerable evidence of a distinct coagulopathy associated with COVID‐19 characterised by elevated D‐dimers and an increased risk of thrombotic events. This comprehensive review summarises the latest evidence from published studies and discusses the implications of the various haematological manifestations of COVID‐19 with a view to guiding clinical management and risk stratification in this rapidly evolving pandemic.

**Novelty Statement**

1. This is the most comprehensive review to date about the haematological aspects of COVID‐19 including the latest evidence of the morphological and clinical manifestations with emphasis on the distinct coagulopathy associated with the disease.
2. This review highlights the paramount importance of haematological parameters such as inflammatory markers, D‐dimer level and absolute lymphocyte count in defining prognostic groups and monitoring response to therapy in COVID‐19.
3. The emphasis on the increased thrombotic risk and the importance of anticoagulation in COVID‐19 is of specific relevance to clinicians.

## 1 INTRODUCTION

Since the first cases of the coronavirus disease 2019 (COVID‐19) were reported in Wuhan, China in December 2019, the disease has rapidly evolved into a pandemic with over 3 million confirmed cases and 200 000 deaths in 185 countries as of April 2020.^1^ COVID‐19 is caused by the novel severe acute respiratory syndrome coronavirus 2 (SARS‐C oV‐2), a betacoronavirus which is transmitted mainly via droplets and contaminated surfaces.^2^, ^3^ Over the past 2 decades, two other severe respiratory diseases, severe acute respiratory syndrome (SARS) and Middle East Respiratory Syndrome (MERS), have been caused by betacoronaviruses, SARS‐CoV and MERS‐CoV, respectively, in humans.^4^ SARS‐CoV‐2 shares 79% sequence identity with SARS‐CoV, the virus responsible for the SARS outbreak 2002‐2004.^5^, ^6^ While much of the pathogenesis of COVID‐19 remains to be unravelled, it is known that SARS‐CoV‐2, like SARS‐CoV, binds to host cells via its receptor, angiotensin converting enzyme 2 (ACE2), which is expressed across a wide range of human cell types including lung type II pneumocytes and the endothelium of blood vessels.^7^, ^8^ The clinical spectrum of COVID‐19 ranges from asymptomatic to severe pneumonia or acute respiratory distress syndrome resulting in respiratory failure and death.^9^, ^10^ While COVID‐19 is considered to be primarily a respiratory infection, there is increasing evidence of multi‐systemic complications of the disease. With more case series being published, a number of haematological manifestations have been now come to light. The key haematological abnormalities of COVID‐19 are summarised in Table 1.

**TABLE 1:** Haematological manifestations of COVID‐19

Lymphopenia^a^
Neutrophilia^a^
Mild thrombocytopenia^a^
Monocytopenia
Elevated LDH^a^
Reactive and plasmacytoid lymphocytes on blood film
Elevated ferritin^a^
**COVID‐19‐associated coagulopathy**
Elevated D‐dimers^a^
Prolonged PT^a^
Prolonged APTT
Elevated fibrinogen

Abbreviations: APTT, activated partial thromboplastin time; LDH, lactate dehydrogenase; PT, prothromin time.

^a^ Features of prognostic significance (see Table S1 for the relevant study data).

## 2 BLOOD COUNT ABNORMALITIES

The most commonly reported blood count abnormality is lymphopenia which occurs in 35%‐83% of patients.^11^, ^12^, ^13^ Lymphopenia is also more frequent and the absolute lymphocyte count (ALC) much lower in severe cases of COVID‐19.^14^, ^15^, ^16^ In one study of haematological parameters in hospitalised COVID‐19 patients in Singapore, the median nadir of the ALC was significantly lower in patients requiring admission to intensive care unit (ICU) (0.4 × 10^9^/L vs 1.2 × 10^9^/L) as was neutrophilia (11.6 × 10^9^/L vs 3.5 × 10^9^/L).^13^ Another study from Wuhan revealed a similar pattern with more severe cases having higher neutrophil (4.3 × 10^9^/L vs 3.2 × 10^9^/L; *P* < .001) counts, lower lymphocytes counts (0.8 1.0 × 10^9^/L vs 1.0 × 10^9^/L; *P* < .001), higher neutrophil‐to‐lymphocyte ratio (5.5 vs 3.2; *P* < .001) as well as lower percentages of monocytes, eosinophils and basophils.^17^ In addition to a significant reduction in both CD4+ and CD8+ T lymphocyte subsets in COVID‐19 patients, severe cases had much lower CD8+ lymphocytes and a subsequent increase positively correlated with improved clinical outcomes.^18^

Mild thrombocytopenia (100‐150 × 10^9^/L) has been reported in up to 20%‐36% of COVID‐19 cases^11^, ^12^, ^13^ however, severe thrombocytopenia (<50 × 10^9^/L) is unusual. In a case series of patients admitted to an intensive care unit in Wuhan, a platelet count of <100 × 10^9^/L was observed in only 5% of patients.^14^ Similar findings were reported in a French cohort study with mild thrombocytopenia identified in about a quarter of COVID‐19 patients on admission to hospital and this was independently predictive of the risk of admission to ICU, mechanical ventilation or death.^19^ Furthermore, data from multiple studies suggest that anaemia is not a prominent feature of COVID‐19 even in severe cases.^11^, ^13^, ^14^ A case series of autoimmune haemolytic anaemia occurring in COVID‐19 patients was recently published but about half of the patients in this study had an underlying haematological disorder known to be associated with autoimmune haemolysis.^20^

## 3 PERIPHERAL BLOOD FILM AND MORPHOLOGICAL FEATURES

**FIGURE 1:**
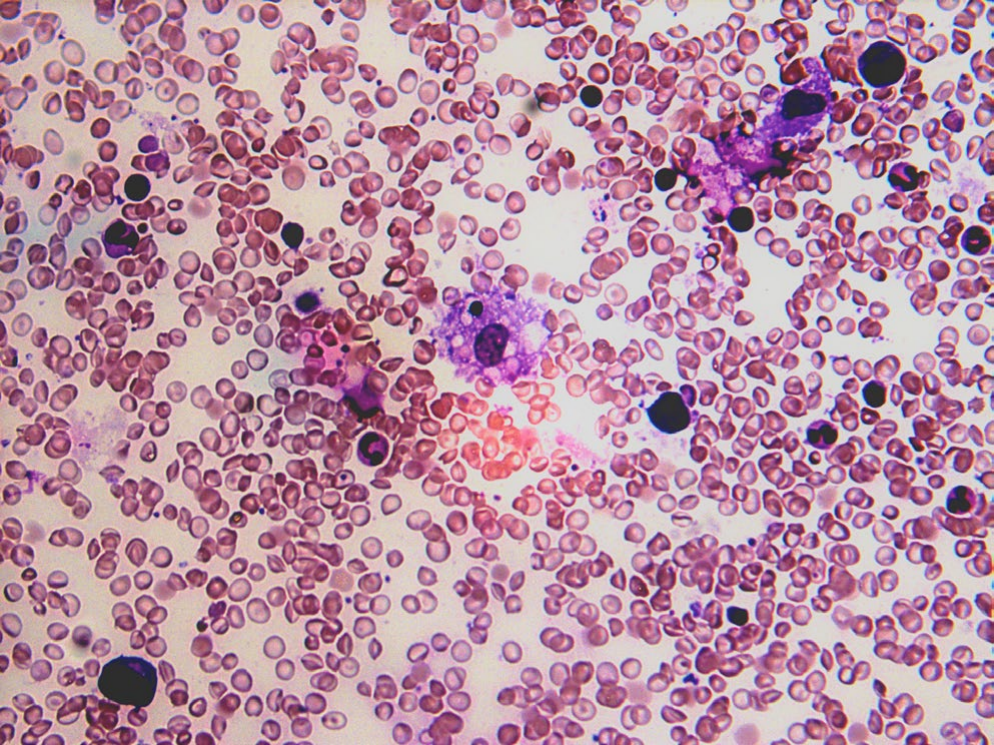
Bone marrow smear showing hemophagocytosis in a 29‐year‐old male patient with severe COVID‐19 and serologic features consistent with hemophagocytic lymphohistiocytosis

Examination of the peripheral blood smear remains a crucial part of the haematological assessment and can often aid the diagnosis of many conditions. Notable features reported in COVID‐19 cases include an increased frequency of reactive and plasmacytoid lymphocytes,^13^, ^21^ significant left‐shifted granulopoiesis with hypergranular, occasionally vacuolated neutrophils^22^ and leucoerythroblastic features.^23^ The presence of schistocytes or red cell fragments has not been reported. Furthermore, from the experience of the authors, bone marrow hemophagocytosis can be a feature of severe COVID‐19 and this has been observed in a small number of patients managed at New Cross Hospital, Wolverhampton, United Kingdom. The bone marrow aspirate from one of the patients is shown in Figure 1. This pathologic finding corroborates recent reports of cases of COVID‐19 meeting the clinical criteria for secondary hemophagocytic lymphohistiocytosis (sHLH).^24^, ^25^

## 4 OTHER BLOOD MARKERS

Alongside other acute‐phase markers such as C‐reactive protein (CRP), procalcitonin and erythrocyte sedimentation rate, an elevated ferritin has been associated with increased mortality in COVID‐19.^17^, ^26^, ^27^ Significant elevation of lactate dehydrogenase has also been observed in patients with severe disease.^13^

## 5 COVID‐19 CYTOKINE STORM AND HAEMATOLOGICAL HYPERINFLAMMATORY SYNDROMES

Observations from the clinical, biochemical and serological manifestations of COVID‐19 strongly support an immunological basis for the severe manifestations of the disease.^27^ The marked elevation of pro‐inflammatory markers such as IL‐1β, IL‐2, IL‐4, IL‐6, IL‐10, TNF‐α and IFNγ frequently seen in severe COVID‐19,^28^, ^29^, ^30^ point to a state of disordered and exaggerated immune response to SARS‐CoV‐2 infection, often referred to as the “cytokine storm”. Several complex pathways have been implicated in the pathogenesis of COVID‐19 cytokine storm including the Renin‐Angiotensin‐Aldosterone system (RAAS), JAK/STAT and Complement activation pathways.^29^, ^31^, ^32^ Furthermore, parallels can be drawn with the cytokine release syndrome (CRS) associated with Chimeric Antigen Receptor (CAR) T‐cell therapy and HLH, which are both states of immune dysregulation and hyperinflammation frequently encountered by haematologists. CRS, thought to be due to T‐cell activation, is characterised by marked elevation of inflammatory markers and cytokines notably IL‐6, fever, hypotension and respiratory insufficiency following the infusion of CAR T cells or other immune therapies.^33^, ^34^ Similarly, HLH is characterised by uncontrolled activation of cytotoxic T lymphocytes, natural killer (NK) cells and macrophages resulting in hypercytokinemia and immune‐mediated organ damage.^35^ The aetiology of HLH may be primary (as a result of inherited genetic mutations) or secondary to other conditions including viral infections and often presents with features of CRS in addition to histological evidence of hemophagocytosis, cytopenias, hyperferritinemia, organomegaly, coagulopathy and multi‐organ failure.^36^ Secondary HLH (sHLH) is probably an under‐recognised feature of severe COVID‐19. A recent case series of 4 autopsies of patients who died from COVID‐19 published as a preprint,^25^ reported histological evidence of haemophagocytosis within pulmonary and hilar lymph nodes in the majority of cases. However, in a study of 40 critically ill COVID‐19 patients admitted to an intensive care unit, only 3 (7.5%) met the cut off for sHLH using the HScore.^24^ This observation was most likely due to the absence of certain cardinal features such as organomegaly and hypofibrinogenemia in the majority of COVID‐19 patients, possibly indicating a distinct mechanism of SARS‐CoV‐2‐associated sHLH. Understanding the link between CRS, HLH and COVID‐19 cytokine storm is crucial because this would expedite the repurposing of therapies for severe COVID‐19. Indeed, early clinical studies have indicated that certain immunomodulatory therapies used in CRS and sHLH such as dexamethasone,^37^ tocilizumab^38^ and anakinra^39^ may be effective in severe COVID‐19.

Given the pivotal role of IL‐6 signalling in CRS and its prognostic significance in COVID‐19,^40^ there has been considerable interest in the therapeutic potential of the IL‐6 receptor antagonist, tocilizumab. Two separate case reports of the use of tocilizumab in acute chest syndrome of Sickle cell disease precipitated by COVID‐19 reported rapid responses in an adult^41^ and a teenager.^42^ Tocilizumab administration also resulted in clinical improvement in a patient with COVID‐19 and multiple myeloma.^43^ A recently published retrospective cohort study of patients treated with tocilizumab, the largest to date, observed a significant reduction in the risk of mechanical ventilation or death in tocilizumab‐treated patients compared with controls.^38^ Several randomised clinical trials of tocilizumab are ongoing and the results of these studies are eagerly awaited.

## 6 COVID‐19‐ASSOCIATED COAGULOPATHY (CAC)

Recent data emerging from the management of patients with COVID‐19 suggests an increased thrombotic tendency. Approximately one‐third of patients with COVID‐19 had CT scan evidence of pulmonary embolism (PE) in a French study.^44^ Notably, two‐thirds of the patients without PE in this cohort also had elevated D‐dimers with a higher cut off value of 2660 µg/L being more predictive of PE in this cohort. A Dutch study of COVID‐19 patients admitted to ICU similarly identified a 31% incidence of thrombotic events including PE, deep vein thrombosis (DVT) and ischaemic strokes despite all patients having received prophylactic anticoagulation.^45^ A retrospective study of COVID‐19 patients admitted to ICU identified DVT in 25% with advanced age, lower lymphocyte counts and elevated D‐dimers being significant risk factors.^46^ The prognostic importance of D‐dimer testing was further demonstrated in a prospective study of hospitalised COVID‐19 patients which revealed that significantly higher D‐dimer and prolonged prothrombin time (PT) were associated with a higher probability of mortality.^47^ In a study investigating prolonged activated partial thromoplastin time (APTT) in patients with COVID‐19, the lupus anticoagulant was detected in 91%.^48^ In addition, markedly elevated Von Willebrand factor (VWF) levels (VWF:antigen‐555%, VWF:activity‐520%) and factor VIII (clotting activity of 369%) in addition to antiphospholipid antibodies were observed in a patient with severe COVID‐19.^49^ Taken together, these findings strongly support the existence of a syndrome of COVID‐19‐associated CAC characterised by derangements in clotting tests (PT and APTT), elevated D‐dimer and an increased thrombotic tendency.

In a study comparing coagulation parameters in hospitalised COVID‐19 patients, 15 (71.4%) of non‐survivors met the International Society on Thrombosis and Haemostasis (ISTH) criteria for overt disseminated intravascular coagulation (DIC) compared with 1 (0.6%) of survivors.^47^ Therefore, the ISTH DIC scoring system which includes platelet count, fibrinogen, D‐dimer and prothrombin time (Table 2) is likely to be a useful prognostic tool for COVID‐19‐associated CAC. The CAC of COVID‐19 appears to be distinct from DIC due to other causes in elevated fibrinogen,^50^ modest prolongation of the APTT and the absence of schistocytes on the blood film.^51^ Interestingly, despite the derangements in coagulation tests, abnormal bleeding is unusual.^52^, ^53^ Furthermore, much of the data for CAC in COVID‐19 has been from hospitalised patients with the severe form of the disease. It is yet unclear but unlikely that patients with mild COVID‐19 are at increased risk of thrombosis.

**TABLE 2:** ISTH Criteria for overt DIC has prognostic significance in COVID‐19‐related coagulopathy (modified from 54)

	Score
Platelet count	
>100	0
50‐100	1
<50	2
Elevated fibrin‐related marker eg D‐dimer	
Not elevated	0
Moderate increase (1‐10× ULN)	1
Severe increase (10× ULN)	2
Prolonged prothrombin time	
<3 s above ULN	0
>3 to <6 s above ULN	1
>6 s above ULN	2
Fibrinogen level	
<1 g/L	0
>1 g/L	1
Total score of ≥5: compatible with overt DIC	

## 7 DISCUSSION

While still not fully understood, the immune response to SARS‐CoV‐2 is thought to be two‐phased; an initial T‐cell mediated adaptive response during the presymptomatic or non‐severe stage which in a proportion of individuals is ineffective, leading to uncontrolled viral replication, triggering an exaggerated innate immune response which results in multi‐organ failure in the most severe cases.^55^, ^56^ The dysregulated secretion of pro‐inflammatory cytokines perpetuates hyperinflammation and is thought to be the basis of the cytokine storm in COVID‐19. This two‐phased model of the immune response would probably account for the lack of efficacy of some immunosuppressive therapies in non‐severe cases of the disease.^37^ Furthermore, ageing is generally associated with diminished adaptive immune function and an exaggerated innate immune response to pathogens, notably a shift towards myelopoiesis and neutrophil accumulation in animal models.^57^, ^58^ These age‐related changes in the immune system may, at least partly, explain the increased mortality of COVID‐19 among the elderly. A lot is still not known about the marked difference between immune responses to SARS‐CoV‐2 between individuals and it is likely that genetic and environmental factors also play a role. Of particular interest is the association between certain ABO blood group genotypes and the likelihood of severe COVID‐19. In a recently published genomewide association study, Blood group A and a few other single nucleotide polymorphisms were associated with an increased risk of COVID‐19‐induced respiratory failure (Blood group O was apparently protective).^59^ Interestingly, blood group A has also been shown to be associated with increased odds of thromoboembolic and cardiovascular events.^60^, ^61^ These findings are hardly surprising because blood group A individuals (as well as other non‐O blood groups) are known to have higher plasma Von Willebrand levels^62^, ^63^ and ABO blood group antigens have innate immune functions.^64^

Currently, the evidence base for the clinical management of COVID‐19 is mostly limited to case series and other relatively small observational studies of hospitalised patients. Despite the limitations, these studies provide useful insight into the manifestations of the disease. Similar to findings in SARS patients,^65^ lymphopenia is the most commonly reported haematological abnormality in COVID‐19 and recent data show that it can be predictive of disease severity. However, as an isolated finding, lymphopenia is not specific for COVID‐19 and is a common finding in the elderly. The predictive utility of lymphopenia is likely to be improved when the trend is considered alongside other parameters particularly the presence of neutrophilia – a higher neutrophil‐lymphocyte ratio. Furthermore, assessments of lymphocyte subsets in COVID‐19 patients have revealed a significant reduction in T and B lymphocytes along with NK cells, more marked among patients with severe disease.^17^, ^28^, ^66^ Of particular interest was the finding of reduced regulatory T cells (Treg) in severe cases.^28^ The precise mechanism of the development of lymphopenia in COVID‐19 is not known but lymphocytes have been shown to express ACE2^67^ and lymphoid cell apoptosis may be a consequence of SARS‐CoV‐2 infection.^68^ Trafficking of lymphocytes away from the peripheral blood to the lungs or other sites of infection may also play a role.^69^ The neutrophilia observed in severe cases of COVID‐19 is most likely a response to the cytokine storm which has been implicated in the most severe manifestations of the disease. Furthermore, significant elevations of acute‐phase markers such as ferritin, CRP and procalcitonin have been associated with mortality from COVID‐19 and these biomarkers are positively correlated with increased pro‐inflammatory cytokines such as Interleukin‐6 and TNF‐α.^26^, ^28^ Therefore, serial monitoring of these markers may facilitate early therapeutic intervention with experimental immunomodulatory agents.

With recent reports of thrombotic complications in patients with COVID‐19, there is increasing recognition of a distinct CAC associated with COVID‐19. The underlying mechanism is likely to be multifactorial including direct endothelial damage from SARS‐CoV‐2 or immune cells,^70^ inflammatory cytokine‐induced activation of the coagulation cascade,^71^ the development of antiphospholipid antibodies and an increase in acute‐phase pro‐coagulants such as factor VIII and fibrinogen. Derangements of coagulation reported among COVID‐19 cases include prolongation of the PT and to a lesser extent, APTT. The relative shortening of the APTT is possibly due to significantly increased levels of Factor VIII. Furthermore, hyperviscosity has been postulated as a possible link between hyperinflammation and CAC following the association of an increased risk of arterial and venous thromboses with plasma viscosity levels greater than 3.5 centipoise (twice the upper limit of normal) in a study of ICU patients with COVID‐19.^72^ However, the hyperviscosity in COVID‐19 is most likely due to the marked elevation of acute‐phase proteins and does not completely explain the severe thrombophilic state which is out of proportion to other conditions associated with comparable degrees of plasma viscosity. Plasma exchange may nevertheless be beneficial as this could theoretically replace most of the elevated circulating cytokines and pro‐coagulant factors. The use of convalescent plasma may, in addition, provide neutralising antibodies against SARS‐CoV‐2 and a small‐scale clinical trial has reported modest but encouraging results in severely ill but not in critical COVID‐19 patients.^73^ The lack of efficacy in the latter group probably reflects the irreversibility of end‐organ damage from hyperinflammation and the relatively minimal contribution from SARS‐CoV‐2 at this stage of the disease.

From a prognostic perspective, D‐dimers appear to be the most useful coagulation parameter as progressive increase in D‐Dimer level is associated with the development of severe disease and in‐hospital mortality.^47^ Therefore, serial monitoring of D‐dimers can be a useful biomarker of clinical severity in patients with COVID‐19.

## 8 MANAGEMENT OF COVID‐19‐ASSOCIATED COAGULOPATHY

The mainstay of the management of CAC is prevention as well as early recognition and treatment of thrombotic events. Regarding the prevention of thrombotic events in hospitalised COVID‐19 patients, evidence from China suggests that prophylactic heparin reduces mortality in high‐risk patients.^52^ Consequently, the recently published ISTH interim guidance recommends prophylactic anticoagulation with low molecular weight heparin (LMWH) for all hospitalised patients with COVID‐19.^74^ LMWH is the preferred anticoagulant as it does not interfere with the PT or APTT and the anti‐inflammatory properties of heparins could indeed provide additional benefits in this setting.^75^ Unfractionated heparin with anti‐Xa monitoring may be a useful alternative given its widespread use in the intensive care setting but close monitoring for the development of heparin‐induced thrombocytopenia is recommended. Furthermore, since CAC is not commonly associated with a bleeding phenotype, anticoagulation is recommended regardless of derangements in the PT or APTT, provided other contraindications are excluded. Moreover, transfusion of blood products such as fresh frozen plasma or cryoprecipitate to correct abnormal clotting parameters in the absence of bleeding is not recommended as this could be detrimental. Cautious transfusion of blood products is however indicated in bleeding patients. Given the histological finding of widespread fibrin deposition consistent with microvascular thrombosis reported in COVID‐19 cases,^76^ clinical trials are needed to evaluate the efficacy of experimental fibrinolytic therapies such as tissue plasminogen activator.^77^

## 9 RECOMMENDATIONS AND CONCLUSIONS

In view of the reported high incidence of thrombotic events despite prophylactic anticoagulation in critically ill patients with COVID‐19,^53^, ^78^, ^79^ there is a strong argument for intensification of anticoagulation in this setting but the benefit of this strategy remains to be determined due to the lack of clinical trial data. While there is currently insufficient evidence to recommend therapeutic anticoagulation in all patients with COVID‐19 in the absence of venous thromboembolism, an individualised risk‐based approach to intensification of anticoagulation may be considered.

In summary, severe COVID‐19 represents a state of immune dysregulation and hyperinflammation which account for the multi‐systemic manifestations, including the haematological anomalies associated with the disease. Therefore, monitoring of haematological parameters, such as the ALC, neutrophil‐to‐lymphocyte ratio and D‐dimers can offer prognostic insight in the management of COVID‐19 and will help with early identification of the high‐risk group of patients requiring more intensive care. In view of the increased thrombotic risk associated with COVID‐19, prophylactic anticoagulation with low molecular weight heparin is recommended for all hospitalised patients with the disease and clinical trials are needed to investigate the role of more intensive anticoagulation and other experimental therapies. In addition, further translational research is needed to fully unravel the pathogenesis of COVID‐19, particularly the host immune response, with a view to developing effective therapies.

## CONFLICT OF INTERESTS

The authors declare no conflict of interests in relation to this article.

## Supporting information

Table S1
