# Supplementary material for: Haematological manifestations of COVID‐19: From cytopenia to coagulopathy
Source: Eur J Haematol. 2020 Aug 31;105(5):540–6. doi: 10.1111/ejh.13491 (PMC7404736; doi:10.1111/ejh.13491)
Supplement: Supplementary file 1 — Table S1 [file EJH-105-540-s001.docx]

| **Hematologic feature** | **Study** | **Study population** | **Findings** |
| --- | --- | --- | --- |
| Lymphopenia | Fan *et al* 2020 | Hospitalised COVID-19 patients in Singapore (n=69) | ALC on admission and nadir significantly lower in patients requiring ICU care |
|  | Qin et al 2020 | COVID-19 patients admitted to a hospital in Wuhan, China (n=452) | Significantly higher NLR and lower ALC in severe cases |
| Neutrophilia | Fan *et al* 2020 | Hospitalised COVID-19 patients in Singapore (n=69) | Peak ANC significantly higher in patients requiring ICU care |
| Thrombocytopenia | Maquet *et al* 2020 | COVID-19 patients admitted to a hospital in France (n=263) | Thrombocytopenia on admission was associated with an increased risk of the composite outcome of ICU admission, mechanical ventilation or death |
| Elevated LDH | Zhou *et al* 2020 | COVID-19 patients admitted to two referral hospitals in Wuhan, China (n=191) | Raised serum LDH was associated with an increased risk of in-hospital mortality |
| Elevated Ferritin | Qin et al 2020 | COVID-19 patients admitted to a hospital in Wuhan, China (n=452) | Significantly higher serum ferritin in severe cases |
|  | Zhou *et al* 2020 | COVID-19 patients admitted to two referral hospitals in Wuhan, China (n=191) | Serum ferritin significantly higher in non-survivors |
| **Elevated D-dimers** | Zhou *et al* 2020 | COVID-19 patients admitted to two referral hospitals in Wuhan, China (n=191) | Marked elevation of D-Dimers in non-survivors |
|  | Cui *et al* 2020 | ICU patients with severe COVID-19 in a hospital in Wuhan, China (n=81) | Significantly higher D-dimers in patients who had VTE |
|  | Tang *et al* 2020 | COVID-19 patients admitted to a hospital in Wuhan, China (n=183) | Significantly higher D-dimers and FDPs in non-survivors |
| **Prolonged PT** | Tang *et al* 2020 | COVID-19 patients admitted to a hospital in Wuhan, China (n=183) | Significantly prolonged prothrombin time in non-survivors |

**SUPPLEMENTARY TABLE**

Haematological manifestations of COVID-19 of prognostic significance.

Abbreviations: ALC-Absolute lymphocyte count, ANC-Absolute neutrophil count, FDP-fibrin degradation products, LDH-lactate dehydrogenase, PT- prothrombin time, NLR-neutrophil-to-lymphocyte ratio.
